# Latent Autoimmune Diabetes in Adults and Metabolic Syndrome—A Mini Review

**DOI:** 10.3389/fendo.2022.913373

**Published:** 2022-06-28

**Authors:** Niansi Pan, Shimei Yang, Xiaohong Niu

**Affiliations:** ^1^ Department of Endocrinology, Changzhi Medical College, Changzhi, China; ^2^ Department of Endocrinology, Changzhi Medical College Affiliated Heji Hospital, Changzhi, China

**Keywords:** Latent Autoimmune Diabetes in Adults, LADA, Metabolic Syndrome, MetS, Obesity, Insulin Resistance, type 2 diabetes mellitus, type 1 diabetes mellitus

## Abstract

Latent autoimmune diabetes in adults (LADA) is a heterogeneous subtype of diabetes characterized by islet cell destruction mediated by islet autoimmunity and insulin resistance. Metabolic syndrome (MetS) is a state in which many risk factors for metabolic and cardiovascular diseases accumulate in an individual. Based on clinical data, this review covers the prevalence of MetS in LADA, focusing on the risk associated with and the role of insulin resistance in the development of LADA from the perspective of inflammatory factors, environmental factors, and the gut microbiota, aiming to improve our understanding of this condition.

## 1 Introduction

In 2019, the World Health Organization classified latent autoimmune diabetes in adults (LADA) as a form of diabetes that shares features of type 1 diabetes mellitus (T1DM) and type 2 diabetes mellitus (T2DM) (1). The incidence of LADA varies among countries, with the highest incidence being concentrated in Europe and China (7%–14%) (2). T1DM, more commonly found in children and adolescents, is characterized by pancreatic β-cell destruction and absolute insulin deficiency. Autoimmunity-mediated β-cell destruction is identified by the presence of insulin autoantibodies (IAAs), glutamic acid decarboxylase antibodies (GADAs), insulinoma-associated antigen-2 autoantibodies (IA-2As), and zinc transporter-8 autoantibodies (ZnT8As). Similar to classic T1DM in adults, LADA is also GADA-positive; however, the clinical features of LADA are more similar to those of metabolic syndrome (MetS), and more islet functions are retained in LADA patients.

T2DM is characterized by a series of metabolic disorders resulting from chronic inflammation and insulin resistance (2). The concept of MetS is constantly developing in the process of preventing the onset of T2DM and its cardiovascular outcome. MetS can be described as the accumulation of several metabolism-related risk factors for cardiovascular disease, including obesity (especially abdominal obesity), diabetes or impaired glucose regulation, dyslipidemia characterized by hypertriglyceridemia, high levels of low-density lipoprotein cholesterol, and hypertension. This accumulation of multiple metabolic abnormalities can significantly increase the morbidity and mortality associated with cardiovascular and cerebrovascular diseases.

This review mainly expounds the epidemiological and clinical characteristics of patients with LADA complicated with MetS and discusses the risk associated with and the role of insulin resistance in the development of LADA focusing on inflammatory factors, environmental factors, and gut microbiota factors, aiming to improve our understanding and, consequently, the diagnosis of LADA.

## 2 Identifying LADA Patients

LADA is a heterogeneous subtype of diabetes mellitus (3) with clinical, metabolic, and immunological features that lie between classic T1DM and T2DM (4). According to the Immunology of Diabetes Society (2005) (5), the diagnostic criteria for LADA is age >30 years in people who present as GADA-positive with youth-onset T2DM diagnosed at between 15 and 29 years of age, which is known as latent autoimmune diabetes in the young (LADY) (6), and positivity for an islet autoantibody. The risk of LADA is related to HLA subtype susceptibility genes for classic T1DM, and islet β cells are often attacked by autoantibodies against both β cells and self-reactive T cells, resulting in impaired insulin secretion. Patients with LADA have a slower islet failure rate and a more extensive clinical phenotype compared with those with classic T1DM (7). LADA can be differentiated from T2DM by the presence of islet autoantibodies or islet antigen-specific T cells. There is evidence to support that LADA may be associated with some of the same environmental risk factors as T2DM, such as being overweight, a lack of exercise, and smoking (8, 9). These factors are known to affect insulin sensitivity, which indicates that insulin resistance may play a key role in the pathogenesis of LADA.

Maturity-onset diabetes of youth (MODY) is a type of young-onset diabetes similar to LADA. MODY is a hereditary (associated with a family history of more than two generations), single-gene, β-cell-function deficiency diabetes accounting for approximately 1%~5% of all cases of diabetes (10). Compared with patients with classic T1DM, MODY patients have higher C-peptide levels and are negative for autoantibodies, and most have a family history of diabetes. Patients with MODY have a lower body mass index (BMI) than those with T2DM and are also diagnosed at a younger age (11), typically before the age of 25. Because of the partial overlap of clinical features, LADA and MODY can easily be confused at the time of diagnosis. The overlap between the two forms is not limited to the onset age. In the progress of the disease, LADA patients do not require insulin therapy within the first 6 months of diagnosis, and MODY patients preserve some islet function 3 to 5 years after diagnosis (5, 12). However, LADA patients with high GADA titers have a faster rate of β-cell failure than MODY patients. One method for distinguishing between the two is the detection of islet autoantibodies (GADAs, ZnT8As, IA-2As, and ICAs) or islet antigen-specific T cells. Patients negative for these factors can then be tested for MODY based on the detection of gene mutation.

## 3 Understanding LADA and MetS Epidemiology

### 3.1 LADA and Obesity

The BMI of LADA patients typically falls within either the underweight or non-obese range. Fourlanos et al. found a BMI <25 kg/m^2^ to be a prominent feature of LADA patients (13). However, other studies have demonstrated that patients with LADA may also suffer from obesity (14, 15). Large-scale surveys conducted in Europe and North America also indicated that the average BMI of LADA patients reached overweight or even obesity levels (16–26) (**Table 1**). Surveys covering China or the Tianjin area of China showed that the average BMI of LADA patients was 25.7 and 23.9 kg/m^2^, respectively, while the overweight or obesity rate was 60.9% in the former and 35.9% in the latter (16, 18). Therefore, regarding diagnostic criteria, being overweight/obese cannot exclude the possibility of being diagnosed as LADA. Meanwhile, differences in BMIs are also found among races. The average BMIs of Chinese and Korean LADA patients were found to be substantially lower than those reported in European studies (for example, 31.4 kg/m^2^ in the ADOPT study) (17, 18, 20). Similar results were obtained in studies from Italy and Korea (27.2 ± 6.2 vs. 23.1 ± 3.7 kg/m^2^) (17, 21). An analysis based on two studies—the ESTRID study (case–control) and the HUNT study from Norway (prospective)—indicated that obesity was associated with an increased risk of LADA (odds ratio [OR] 2.93, 95% CI 2.17–3.97 in the ESTRID study; hazard ratio [HR] 6.07, 95% CI 3.76–9.78 for the HUNT study) (27). These results support that obesity increases the risk of LADA.

**Table 1:** The mean body mass index of LADA.

Study	Country	Type of study	Number of sample size	Age range, yr	Ab	Mean BMI (kg/m^2^)
Tianjin (2005)	China (Tianjin)	Population based	8,109	≥15	GAD	25.7 ± 3.4
Lee et al. (17)	Korea	Clinical based	1,370	47–62	GAD and/or IA-2	23.1 ± 3.7
LADA China (2013)	China	Multicenter, clinical based	5,324	≥20	GAD	23.9 ± 3.7
UKPDS 25 (1997)	United Kingdom	Clinical based	3,672	25–65	GAD and/or ICA	29.5 ± 5.5
ADOPT (2004)	USA, Europe	Multicenter, clinical based	4,357	30–75	GAD and/or IA-2	31.4 ± 0.466
NIRAD (2007)	Italy	Multicenter, clinical based	4,250	30–75	GAD and/or IA-2	27 ± 5.16
Action LADA (2009)	Europe	Multicenter, clinical based	6,810	30–70	GAD and/or IA-2, ZnT8	27.2 ± 6.2
Maioli et al. (23)	Italy (Sardinia)	Clinical based	5,568	35–70	GAD	27.7 ± 4.8
Hawa et al. (24)	Europe	Multicenter, clinical based	2,838	40–75	GAD and/or IA-2, ZnT8	27.3 ± 3.6
Maddaloni et al. (25)	United Arab Emirates	Clinical based	17,072	30–70	GAD and/or IA-2	30.7 ± 6.2
Lofvenborg et al. (26)	Swedish	Population based	2,125	≥35	GAD	27.7 ± 5.2

LADA, latent autoimmune diabetes in adults; GAD, glutamic acid decarboxylase; ICA, islet cell; IA-2, protein tyrosine phosphatase; ZnT8, islet-specific zinc transporter isoform 8.

Some studies have suggested that LADA is associated with a relatively low risk of MetS (17, 28). The degree of LADA complicated with MetS was reported to be negatively correlated with the type and quantity of islet autoantibodies. The clinical phenotype of LADA patients with more types of antibodies is similar to that of patients with T1DM. Patients with LADA double-positive for GADAs and islet cell antibodies (ICAs) have a smaller waist circumference, lower blood pressure, and lower prevalence of MetS than those single-positive for GADAs (14, 15). In addition, compared with patients double-positive or single-positive for GADAs and/or ICAs, those triple-positive for GADAs, ICAs, and IA-2As have a smaller waist circumference and a lower prevalence of hypertension and MetS. However, a different study found that only higher GADA titers were likely to be associated with a lower incidence of MetS (29).

The prevalence of MetS in patients with LADA was reported to range from 41.9% to 74.1% in Asia and Europe (17, 18, 20–22, 24, 30), depending on the diagnostic criteria for MetS (31–33) (**Table 2**). A multicenter survey in 2013 found that the prevalence of MetS was 75.6% in patients with T2DM and 62% in those with LADA (18). A more recent study reported the prevalence of MetS to be 68.1% in patients with T2DM and 44.3% in those with LADA, with patients with T1DM having the lowest prevalence, at 34.2% (30). This study demonstrated that MetS was highly prevalent in patients newly diagnosed with diabetes in China and was present in more than one-third of patients with autoimmune diabetes. Moreover, the prevalence of MetS in the LADA population was slightly lower than that in the T2DM population, but significantly higher than that in patients with T1DM. Data from the Europe Action LADA study showed that the prevalence of MetS in LADA and T1DM patients was 41.9% and 31.9%, respectively, which was markedly different from that observed in T2DM patients (88.8%) (22). Thus, in Europe, the prevalence of MetS in LADA patients was similar to that of T1DM patients and did not differ from that of the normal control population; however, it was substantially lower than that in patients with T2DM.

**Table 2:** The prevalence of MetS in T2DM, LADA, and T1DM.

Study	Country	Type of study	Number of sample size	Age range, yr	Definitions of MetS	MetS (%)			Mean BMI(kg/m^2^)(mean ± SD)	Overweight or obesity(%)	Low HDL(%)	HighTG(%)	High BP(%)	Waist circumference(%)
						T2D	LADA	T1D						
Lee et al. (17)	Korea	Clinical based	1,370	47–62	NCEP ATP III	72.9	47.1	n/d	23.1 ± 3.7	n/d	n/d	n/d	n/d	n/d
LADA China study (2013)	China	Multicenter, clinical based	5,324	≥30	NCEP ATP III	75.6	62.0	n/d	23.9 ± 3.7	35.9	62.4		34.1	n/d
Xia Li et al. (30)	China	Multicenter, clinical based	15,492	≥30	CDS (2017)	68.1	44.3	34.2	n/d	n/d	29.3	33.9	47.9	31.7
ADOPT (2004)	USA, Europe	Multicenter, clinical based	4,357	30–75	NCEP ATP III	83.7	74.1	n/d	31.4 ± 0.5	n/d	n/d	n/d	n/d	n/d
NIRAD (2007)	Italy	Multicenter, clinical based	4,250	30–75	NCEP ATP III	67.6	58.6	n/d	27.0 ± 5.2	n/d	n/d	n/d	n/d	n/d
Action LADA 3 (2009)	Europe	Multicenter, clinical based	2,011	30–70	NCEP ATP III	88.8	41.9	31.9	n/d	n/d	n/d	n/d	n/d	n/d
Hawa et al. (24)	Europe	Multicenter, clinical based	2,838	40–75	IDF	80.4	63.6	n/d	27.3 ± 3.6	n/d	n/d	n/d	n/d	67.6

Ab, antibody; ATP, National Cholesterol Education Program Adult Treatment Panel III; CDS, Chinese Diabetes Society; IDF, International Diabetes Federation; BP, blood pressure; HDL, high-density lipoprotein; n/d, no data; MetS, metabolic syndrome; T2DM, type 2 diabetes mellitus; LADA, latent autoimmune diabetes in adults; T1DM, type 1 diabetes mellitus; TG, triglycerides.

There is a difference between China and European countries in the proportion of LADA patients complicated with MetS, which may be partly attributed to the “bimodal distribution” characteristics of GADA titers (21). LADA can be subdivided into LADA-type 1 and LADA-type 2 according to the GADA titer. The initial GADA titer determines the persistence of autoimmunity and the different degrees of disease progression (34, 35). LADA-type 1 is always associated with a higher antibody titer, a greater extent of β-cell function impairment, a higher insulin utilization rate, lower overweight and obesity rates, and a lower prevalence of MetS. LADA-type 2 is characterized by a lower antibody titer and a metabolic phenotype and β-cell function impairment similar to that found in T2DM patients. Meanwhile, the overweight/obesity ratio is also similar to that of T2DM patients, and most LADA-type 2 cases are associated with MetS. Notably, however, patients with higher GADA titers in China account for approximately 25% of the total number of LADA patients (18), while in Italian studies the proportion was reported to reach almost 50% (21).

Different types of diabetes mellitus are often associated with specific combinations of MetS characteristics (30). The most frequent combination in T2DM is hypertension plus large waist circumference, hypertriglyceridemia plus low HDL-C is the most common combination in T1DM, while the most common type in LADA is hypertension plus large waist circumference plus hypertriglyceridemia. These observations support that LADA diagnosis cannot be excluded in patients newly diagnosed with diabetes complicated with MetS or expressing MetS characteristics and that patients should be assessed for the presence of autoantibodies for early and correct typing. The blood pressure and blood lipid levels of LADA patients are lower than those of T2DM patients but higher than those of T1DM patients with a similar course of disease, which may be related to the early-onset characteristics of T1DM (36). Subjects with LADY are less likely to have MetS compared with those with T2DM (27.6% vs. 59.4%) (6). With increasing age, the prevalence of MetS increases in LADA patients, and LADA inclines to show a similar prevalence of MetS to T2DM. A study by Zhou et al. (37) found that up to 59.2% of LADA patients ≥60 years old were complicated with MetS, while in T2DM patients of the same age group, the proportion was 67.6%; the difference was not statistically significant. The GADA titer and the fasting C-peptide level were respectively stratified, with the results revealing that elderly LADA patients with lower GADA titers have more MetS components, while those with higher C-peptide levels tend to display more MetS components or have a higher prevalence of MetS, implying that LADA patients with different age phases, GADA titers, and C-peptide levels should be treated differently.

## 4 Understanding LADA and MetS Pathogenesis

### 4.1 Insulin Resistance Plays an Important Role in the Onset of LADA

Although insulin resistance is thought to underlie the pathophysiology of T2DM and to be the core of MetS, the role of insulin resistance in the onset of LADA remains unclear. Clinical studies have indicated that the degree of insulin resistance in LADA patients is lower than that in patients with T2DM but comparable to that in patients with T1DM (20). Several studies have reported that the incidence of insulin resistance in LADA and antibody-negative T2DM patients is higher than that in normal controls (38, 39). Employing the Homeostasis Model Assessment for Insulin Resistance (HOMA-IR) and the Quantitative Insulin Sensitivity Check Index for Insulin Resistance (QUICKI-IR), Chiu et al. (40, 41) found that the degree of insulin resistance in LADA patients was similar to that in T2DM patients. In a different study, the risk of MetS in LADA was found to be higher than that for T1DM, and this increased risk was associated with greater insulin resistance (30). This suggested that insulin resistance is also crucial to LADA pathophysiology and provides a basis for the employment of insulin sensitizers such as thiazolidinedione in the treatment of LADA.

Zhou et al. (37) found that, like T2DM patients, elderly LADA patients (≥60 years of age) also have a higher level of insulin resistance than young (<60 years old) LADA patients. LADA may be accompanied by non-alcoholic fatty liver disease (NAFLD), and such patients have more severe metabolic disorders, greater insulin resistance, and a higher prevalence of MetS. Moreover, there is an independent correlation between fasting C-peptide levels and NAFLD in LADA patients (42).

### 4.2 Inflammatory Cytokines, Environmental Factors, and the Gut Microbiota Related to Insulin Resistance Play an Important Role in LADA Pathogenesis

#### 4.2.1 Inflammatory Cytokines

T2DM can be effectively predicted by the presence of various inflammatory markers (43). Furthermore, chronic inflammation is considered a key factor in the pathogenesis of T2DM and the development of insulin resistance. Activation of the acute phase response, such as an increase in the levels of C-reactive protein (C-RP) and systemic inflammation, plays a fundamental role in the etiology of T2DM and MetS. Systemic inflammation is linked to the presence of a variety of plasma proteins and pro-inflammatory cytokines (such as C-RP and TNF-α). However, there is also evidence to support the involvement of systemic inflammation in LADA.

Studies from Europe (European Action LADA) and Asia have reported that the levels of inflammatory cytokines, such as high-sensitivity C-RP (hs-CRP), IL-6, serum adhesion molecules (soluble VCAM-1, soluble ICAM-1, and soluble E-selectin), and chemokines (CCL2, CCL3, and CCL4), in LADA patients were similar to those of patients with classic T1DM, lower than those of antibody-negative T2DM patients, and higher than those of normal controls (44, 45). The LADA China study found that, compared with people with normal glucose tolerance, after adjusting for gender, age, and BMI levels, IL-6 and LCN2 concentrations were increased in patients with three types of diabetes (T1DM, LADA, and T2DM). In LADA patients, the levels of hs-CRP and adiponectin were also increased, while in T1DM patients the adiponectin level was increased, but not the hs-CRP level. The study also noted that the GADA titer was positively correlated with the level of adiponectin and negatively correlated with that of hs-CRP. Among the LADA subjects, the hs-CRP level was also found to be closely related to the BMI (18). Low-grade markers of inflammation (hs-CRP levels and the leukocyte count), obesity (adiponectin levels), and immunity (soluble TNF-α receptor 2 [sTNFRII] levels) can help distinguish among LADA, typical adult T1DM, and T2DM. The sTNFRII and hs-CRP levels were respectively correlated with an increased risk of LADA and typical adult T1DM. After stepwise adjustment for multiple variables, increased sTNFRII levels persisted as a predictor for higher risk of LADA compared with classic adult-onset T1DM (46).

These observations suggest that there may be a complex relationship between the type of diabetes and cytokine levels. Systemic inflammation, which is closely related to MetS, also plays a role in LADA. However, whether chronic, low-grade inflammation is related to the gradual decline in pancreatic islet function in LADA patients, and thus has the potential to become a key target of LADA intervention therapy, remains unknown.

#### 4.2.2 Environmental Factors

Environmental factors influence T2DM pathogenesis primarily by increasing the level of insulin resistance (47). Numerous studies have reported that a correlation also exists between LADA and environmental factors. After adjusting for age, gender, BMI, physical activity, smoking, and education, men with sleep disorders were at increased risk of autoimmune diabetes (HR 1.83, 95% CI 1.05–3.20), while impaired mental health was correlated with an increased risk of autoimmune diabetes in women (48). Based on data from three surveys by the Nord-Trump Health Research Center, Rasouli et al. concluded that smoking could reduce the incidence of autoimmune diabetes (49). However, a subsequent study (the ESTRID study) conducted by the same author involving a larger cohort found that heavy smoking could increase the risk of LADA (OR 1.37, 95% CI: 1.02–1.84). Furthermore, among LADA patients, HOMA-IR and HOMA-β values were higher in heavy smokers than in non-smokers, whereas the GADA level was lower (50). The authors speculated that the protective effect of smoking on LADA may be concealed by an increase in smoking-induced insulin resistance, similar to the pro-diabetic effect observed in T2DM patients. They also reported that coffee intake was positively correlated with the risk of LADA among high-risk HLA genotype carriers (51). Daily intake of 200 ml of sweetened drinks was associated with increased LADA risk (26). Increasing age, obesity, and a lack of exercise are all important risk factors for LADA (OR: 1.15, 95% CI: 1.02–1.29) (52), further revealing the important role of insulin resistance in the pathogenesis of this condition.

#### 4.2.3 Gut Microbiota

Gut dysbiosis may be a risk factor for the occurrence and development of many human diseases. T2DM and T1DM have mild or moderate gut microbiota imbalance (53–55). Gut dysbiosis may be accompanied by an increase or decrease in the production of gut microbiota-derived metabolites, such as branched-chain amino acids, short-chain fatty acids, and indoles. Some gut microbiota members can enhance the degree of insulin resistance through the biosynthesis of branched-chain amino acids (56). The evaluation of insulin sensitivity using the hyperinsulinemic–euglycemic clamp in patients newly diagnosed with T1DM and T2DM highlighted differences in plasma metabonomics between the two groups of patients. However, alanine, α-amino-adipic acid, isoleucine, and stearic acid were found to be negatively correlated with insulin sensitivity in both T2DM and T1DM and thus could be used as biomarkers for insulin sensitivity (57). In a survey of 531 Finnish men with MetS (METSIM study), a correlation was detected between the gut microbiota and the levels of various metabolites in fasting serum, such as trimethylamine oxide, fatty acids, amino acids, and lipids (58). Gut microbiota-derived metabolites can enter the blood circulation through a compromised intestinal barrier, resulting in immune or inflammatory responses. Gut dysbiosis may influence the metabolic phenotype. A 4-year longitudinal study of 106 patients with prediabetes in the United States showed that there were differences in the levels of inflammatory cytokines as well as in the gut microbiota and their metabolites under different blood glucose levels (59). Compared with T1DM and T2DM patients and with the normal population, patients with LADA display distinct gut microbiota and metabolite characteristics. A recent study found that a decrease in the abundance of short-chain fatty acid-producing bacteria and the contents of gut microbiota-derived metabolites such as branched-chain amino acids (valine, leucine, and isoleucine) and aromatic amino acids (tyrosine and phenylalanine) was associated with islet autoantibodies, glucose metabolism, islet function, and inflammatory cytokines (IL-6, IL-1β, TNF-α) (60). Whether the gut microbiota and derived metabolites can serve as targets for the regulation of the metabolism of LADA patients merits further exploration.

## 5 Conclusion

Modern unhealthy lifestyles and diets are likely to lead to an increase in the number of patients with obesity and MetS. Accordingly, there will be more LADA patients with insulin resistance, and pre-LADA patients whose islet function has been damaged to some extent may be more easily induced by insulin resistance, which is also expected to increase (45). Therefore, explaining the role of insulin resistance in the onset of LADA and attaching importance to the clinical manifestations and treatment of MetS in LADA patients will further our understanding of LADA and the ability to diagnose and treat this condition.

## Author Contributions

NP wrote the manuscript. NP and SY performed the literature review and revised the manuscript. XN provided ideas and developed the protocol for the review. All authors contributed to the article and approved the submitted version.

## Funding

This study was supported by the “Four batches” Innovation Plan Project of Developing Medicine Through Science and Technology of Shanxi Provincial Health and Wellness Committee (2020XM25).

## Conflict of Interest

All authors declare that the research was conducted in the absence of any commercial or financial relationships that could be construed as a potential conflict of interest.

## Publisher’s Note

All claims expressed in this article are solely those of the authors and do not necessarily represent those of their affiliated organizations, or those of the publisher, the editors and the reviewers. Any product that may be evaluated in this article, or claim that may be made by its manufacturer, is not guaranteed or endorsed by the publisher.
